# A descriptive analysis of scoring patterns on clinically relevant questionnaires in 26 adults with diagnosed muscle dysmorphia

**DOI:** 10.1002/erv.3001

**Published:** 2023-06-19

**Authors:** Jordan A. Martenstyn, Sarah Maguire, Scott Griffiths

**Affiliations:** ^1^ Clinical Psychology Unit School of Psychology The University of Sydney Camperdown NSW Australia; ^2^ InsideOut Institute for Eating Disorders Charles Perkins Centre The University of Sydney Camperdown NSW Australia; ^3^ Sydney Local Health District NSW Health Sydney NSW Australia; ^4^ Melbourne School of Psychological Sciences Faculty of Medicine Dentistry and Health Sciences The University of Melbourne Parkville Australia

**Keywords:** bigorexia, compulsive exercise, disordered eating, muscle dysmorphia, questionnaire

## Abstract

**Objective:**

Few studies have recruited adults with a formal diagnosis of muscle dysmorphia (MD), a psychological illness defined by preoccupation that one lacks muscularity and/or leanness, combined with significant functional impairment. This study presented descriptive statistics for a range of clinically relevant questionnaires in one of the largest samples of adults with confirmed diagnoses of MD.

**Method:**

We recruited 29 adults who met diagnostic criteria for MD as determined by semi‐structured diagnostic interviews and administered a battery of questionnaires assessing MD symptomology, compulsive exercise, and disordered eating. Descriptive statistics were calculated for both total and subscale scores for each questionnaire. Raincloud plots are included to show the distribution of total scores.

**Results:**

Of the 29 participants, 26 completed all questionnaires. Mean scores were high across all questionnaires and broadly consistent with results in past studies that also recruited a clinical MD sample. Most participants scored above proposed clinical cut‐off scores for questionnaires assessing compulsive exercise and disordered eating.

**Conclusions:**

This study adds to the small body of published questionnaire data in clinical MD samples. We stress that questionnaire scores should not be used alone to infer the presence of MD, but could be considered as a useful adjunct to a comprehensive clinical interview.

## Key Points

- We have limited understanding of how people with a formal diagnosis of muscle dysmorphia (MD) present clinically and score on questionnaires assessing core cognitions and behaviours involved in the disorder.
- People with MD score high on questionnaires assessing MD symptomology, compulsive exercise, and disordered eating.
- Participant scores exceeded proposed clinical cut‐off scores to a much greater degree on instruments assessing compulsive exercise than disordered eating.

## 1 INTRODUCTION AND AIMS

Muscle dysmorphia (MD) is a psychological illness included in the latest edition of the Diagnostic and Statistical Manual of Mental Disorders (DSM‐5) as a specifier of body dysmorphic disorder, which falls under the broader category of obsessive‐compulsive and related disorders (American Psychiatric Association, 2013). MD is defined by excessive preoccupation that one lacks muscularity and/or leanness, strict exercise and muscularity‐oriented diet practices that support the attainment of appearance goals, compulsive mirror checking, frequent avoidance of public body exposure, and significant functional impairment (Martenstyn, Maguire, et al., 2022a; Martenstyn, Maguire, et al., 2022b; Pope et al., 1997). People with MD regard themselves as too small and not muscular enough, despite being substantially more muscular than the average person (Olivardia et al., 2000).

Although the first scientific account of MD was published three decades ago (Pope et al., 1993), there remain significant gaps in the MD literature. First, most MD studies recruited subclinical samples of either university students or regular gym‐goers who want to gain muscle, but lack the degree of body dissatisfaction and functional impairment reported in adults with a formal diagnosis of MD (Cooper et al., 2020; Sandgren & Lavallee, 2018). As such, current understanding of MD is largely based on findings from research conducted with *subclinical* rather than *clinical* MD populations as assessed via diagnostic interview (Rodgers & Murray, 2022). Second, self‐report questionnaires are commonly used in MD research to infer MD diagnostic status and/or the severity of MD symptoms, notwithstanding that clinical norms have not been developed for any MD‐specific questionnaires (Cooper et al., 2020; Prnjak & Murray, 2021; Sandgren & Lavallee, 2018). This lies in stark contrast to related illness groups (e.g., eating disorders) where specific cut‐off scores have been validated for common questionnaires, such as the Eating Disorder Examination‐Questionnaire (EDE‐Q; Fairburn & Beglin, 1994). Thus, the use of questionnaires to determine MD diagnostic status and/or severity is highly problematic until more research is conducted with clinical MD samples to develop reliable clinical norms (Prnjak & Murray, 2021).

Our review of the literature indicates that only 10 studies have reported questionnaire data from a clinical MD sample, with large variations in the amount of published data between questionnaires (see Table 1). For example, five studies (two cross‐sectional descriptive and three case reports) published data on the Muscle Dysmorphic Disorder Inventory (MDDI; Hildebrandt et al., 2004), while only one study published data on the Muscle Appearance Satisfaction Scale (MASS; Mayville et al., 2002). We recently conducted a qualitative study investigating the phenomenology of MD in 29 adults with clinical MD as confirmed via diagnostic interview (Martenstyn, Maguire, et al., 2022a, 2022b). Given the limited amount of questionnaire data published in clinical MD samples, we also administered a battery of questionnaires assessing MD symptomology, compulsive exercise, and disordered eating. Wherever possible, we compared descriptive statistics for questionnaires administered in our study with those used in prior research that also recruited adults with diagnosed MD. We acknowledge that the questionnaires administered in this study were not exhaustive in covering the entire gamut of MD symptoms and behaviours. Nonetheless, our questionnaire data—in conjunction with the associated literature review—provides a useful reference for clinicians and researchers seeking to better understand the response patterns of adults with diagnosed MD on questionnaires clinically relevant to the disorder.

**TABLE 1:** Questionnaire data reported in past studies that recruited a clinical MD sample (organised by questionnaire).

	MD group			Non‐MD/general BDD control group			Sig difference versus control group?
	*n*	Mean	SD	*n*	Mean	SD	
Muscle dysmorphia instruments							
Muscle dysmorphic disorder inventory (MDDI; Hildebrandt et al., 2004)							
Murray, Rieger, et al. (2012)—drive for size subscale^a^	21	20.57	3.54	15	11.00	6.44	Yes (higher), *p* < 0.017
Murray and Griffiths (2015)—drive for size subscale^a^ ^,^ ^b^	1	22.00	n/a	n/a	n/a	n/a	n/a
Murray, Maguire, et al. (2012)—drive for size subscale^a^	1	20.00	n/a	n/a	n/a	n/a	n/a
Murray et al. (2011) ‐ drive for size subscale^a^ ^,^ ^c^	1	20.00	n/a	n/a	n/a	n/a	n/a
Murray, Rieger, et al. (2012)—appearance intolerance subscale^a^	21	15.43	2.52	15	6.87	3.23	Yes (higher), *p* < 0.017
Murray and Griffiths (2015)—appearance intolerance subscale^a^ ^,^ ^b^	1	18.00	n/a	n/a	n/a	n/a	n/a
Murray, Maguire, et al. (2012)—appearance intolerance subscale^a^	1	16.00	n/a	n/a	n/a	n/a	n/a
Murray et al. (2011)—appearance intolerance subscale^a^ ^,^ ^c^	1	16.00	n/a	n/a	n/a	n/a	n/a
Murray, Rieger, et al. (2012)—functional impairment subscale^a^	21	16.00	3.81	15	6.60	3.92	Yes (higher), *p* < 0.017
Cafri et al. (2008)—functional impairment subscale^a^	15	21.67	3.48	36	13.44	3.38	Yes (higher), *p* < 0.001
Murray and Griffiths (2015)—functional impairment subscale^a^ ^,^ ^b^	1	19.00	n/a	n/a	n/a	n/a	n/a
Murray, Maguire, et al. (2012)—functional impairment subscale^a^	1	15.00	n/a	n/a	n/a	n/a	n/a
Murray et al. (2011)—functional impairment subscale^a^ ^,^ ^c^	1	16.00	n/a	n/a	n/a	n/a	n/a
Murray, Rieger, et al. (2012)—total score^a^	21	52.00	6.72	15	24.47	12.73	Yes (higher), *p* < 0.017
Blashill et al. (2020)—total score^a^	15	45.60	6.50	15	34.59	6.50	Yes (higher), *p* < 0.001^e^
Murray and Griffiths (2015)—total score^a^ ^,^ ^b^	1	59.00	n/a	n/a	n/a	n/a	n/a
Murray, Maguire, et al. (2012)—total score^a^	1	51.00	n/a	n/a	n/a	n/a	n/a
Murray et al. (2011)—total score^a^ ^,^ ^c^	1	52.00	n/a	n/a	n/a	n/a	n/a
Muscle appearance satisfaction scale (MASS; Mayville et al., 2002)							
Cafri et al. (2008)—bodybuilding dependence subscale^a^	15	26.07	3.63	36	19.53	5.56	Yes (higher), *p* < 0.001
Cafri et al. (2008)—muscle checking subscale^a^	15	20.13	5.18	36	13.67	5.61	Yes (higher), *p* < 0.001
Cafri et al. (2008)—substance use subscale^a^	15	16.53	7.31	36	12.25	4.02	No, *p* = 0.05
Cafri et al. (2008)—injury risk subscale^a^	15	13.87	5.14	36	11.33	3.55	No, *p* = 0.05
Cafri et al. (2008)—muscle satisfaction subscale^a^	15	15.80	3.55	36	12.36	4.10	Yes (higher), *p* < 0.01
Muscle dysmorphia inventory (MDI; Rhea et al., 2004)							
Waldorf et al. (2019)—size symmetry subscale^a^	24	22.29	4.39	24	12.75	4.00	Yes (higher), *p* < 0.001
Waldorf et al. (2019)—exercise dependence subscale^a^	24	17.58	2.50	24	11.96	3.00	Yes (higher), *p* < 0.001
Waldorf et al. (2019)—physique protection subscale^a^	24	17.38	4.98	24	9.04	2.49	Yes (higher), *p* < 0.001
Waldorf et al. (2019)—diet subscale^a^	24	20.04	3.20	24	13.04	5.62	Yes (higher), *p* < 0.001
Waldorf et al. (2019)—supplement use subscale^a^	24	15.50	3.32	24	8.13	3.55	Yes (higher), *p* < 0.001
Compulsive exercise instruments							
Compulsive exercise test (CET; Taranis et al., 2011)							
Murray, Rieger, et al. (2012)—avoidance and rule‐driven subscale^a^	21	31.52	6.45	15	7.33	10.37	Yes (higher), *p* < 0.017
Murray, Rieger, et al. (2012)—weight control subscale^a^	21	11.62	4.12	15	3.01	5.79	Yes (higher), *p* < 0.017
Murray, Rieger, et al. (2012)—mood improvement subscale^a^	21	20.76	3.52	15	4.13	7.42	Yes (higher), *p* < 0.017
Murray, Rieger, et al. (2012)—lack of exercise enjoyment subscale^a^	21	2.48	3.36	15	4.33	2.66	Yes (lower), *p* < 0.017
Murray, Rieger, et al. (2012)—exercise rigidity subscale^a^	21	11.86	2.33	15	5.13	5.19	Yes (higher), *p* < 0.017
Murray, Rieger, et al. (2012)—total score^a^	21	78.19	10.28	15	34.40	21.77	Yes (higher), *p* < 0.017
Eating psychopathology instruments							
Eating disorder examination questionnaire (EDE‐Q 6.0; Fairburn & Beglin, 2003)							
Murray, Rieger, et al. (2012)—dietary restraint subscale^a^	21	14.19	4.72	15	4.73	5.84	Yes (higher), *p* < 0.017
Murray, Rieger, et al. (2012)—eating concern subscale^a^	21	6.24	6.22	15	1.40	1.99	No, *p* > 0.05
Murray, Rieger, et al. (2012)—weight concern subscale^a^	21	14.86	6.29	15	4.01	4.32	Yes (higher), *p* < 0.017
Murray, Rieger, et al. (2012)—shape concern subscale^a^	21	28.62	11.96	15	9.07	9.57	Yes (higher), *p* < 0.017
Waldorf et al. (2019)—total score^d^	24	1.98	0.85	24	0.54	0.48	Yes (higher), *p* < 0.001
Murray, Rieger, et al. (2012)—total score^a^	21	63.81	22.91	15	19.27	18.13	Yes (higher), *p* < 0.017
Eating disorder examination questionnaire (EDE‐Q 6.0; Fairburn & Beglin, 2003)—with modifications to be more applicable to males							
Murray, Rieger, et al. (2012)—dietary restraint subscale^a^	21	23.19	6.15	15	8.27	8.96	Yes (higher), *p* < 0.017
Murray, Rieger, et al. (2012)—eating concern subscale^a^	21	7.24	6.11	15	1.67	2.55	Yes (higher), *p* < 0.017
Murray, Rieger, et al. (2012)—weight concern subscale^a^	21	19.14	5.88	15	6.40	6.60	Yes (higher), *p* < 0.017
Murray, Rieger, et al. (2012)—shape concern subscale^a^	21	33.10	9.40	15	12.80	11.22	Yes (higher), *p* < 0.017
Murray, Rieger, et al. (2012)—total score^a^	21	79.10	25.32	15	29.80	25.45	Yes (higher), *p* < 0.017
Eating disorder examination (EDE; Fairburn & Cooper, 2003)							
Murray, Maguire, et al. (2012)—dietary restraint subscale^d^	1	2.40	n/a	n/a	n/a	n/a	n/a
Murray, Maguire, et al. (2012)—eating concern subscale^d^	1	0.60	n/a	n/a	n/a	n/a	n/a
Murray, Maguire, et al. (2012)—weight concern subscale^d^	1	1.60	n/a	n/a	n/a	n/a	n/a
Murray, Maguire, et al. (2012)—shape concern subscale^d^	1	4.00	n/a	n/a	n/a	n/a	n/a
Murray, Maguire, et al. (2012)—total score^d^	1	2.20	n/a	n/a	n/a	n/a	n/a
Eating disorders inventory (EDI; Garner et al., 1983)							
Olivardia et al. (2000)—total score^a^	24	44.30	26.00	30	21.90	12.00	Yes (higher), *p* < 0.001
General body dysmorphia instruments							
Body dysmorphic disorder modification of the Yale Brown obsessive‐compulsive scale (BDD‐YBOCS; Phillips et al., 1997)							
Blashill et al. (2020)—total score^a^	15	27.24	5.53	15	23.69	4.68	Yes, *p* < 0.05^e^
Cafri et al. (2008)—total score^a^	15	29.60	6.91	36	9.25	5.98	Yes (higher), *p* < 0.001
Pope et al. (2005)—total score^a^	14	27.30	10.10	49	24.70	11.90	No, *p* > 0.05^e^
Dysmorphic concern questionnaire (DCQ; Oosthuizen et al., 1998)							
Waldorf et al. (2019)—total score^a^	24	8.58	3.53	24	4.17	2.43	Yes (higher), *p* < 0.001
General psychopathology instruments							
Depression anxiety stress Scale‐21 (DASS‐21; Lovibond & Lovibond, 1995)^g^							
Waldorf et al. (2019)—depression subscale^a^	24	4.92	4.12	24	1.46	1.56	Yes (higher), *p* < 0.01
Waldorf et al. (2019)—stress subscale^a^	24	5.66	4.32	24	1.71	1.99	Yes (higher), *p* < 0.001
Short form quality of life enjoyment and satisfaction questionnaire (Q‐LES‐Q; Endicott et al., 1993)							
Pope et al. (2005)—total score^a^	14	42.10	20.60	49	56.80	18.30	Yes (lower), *p* < 0.05^e^
36‐Item short form health survey (SF‐36; Ware & Sherbourne, 1992)							
Pope et al. (2005)—mental health subscale^a^	14	27.30	21.50	49	47.90	23.20	Yes (lower), *p* < 0.01^e^
Pope et al. (2005)—role emotional subscale^a^	14	25.00	37.90	49	37.00	42.90	No, *p* > 0.05^e^
Pope et al. (2005)—social functioning subscale^a^	14	36.50	21.00	49	50.50	28.10	No, *p* > 0.05^e^
Self‐esteem instruments							
Body‐esteem scale for adolescents and adults (BESAA; Mendelson et al., 2001)							
Rodrigue et al. (2018)—appearance subscale^d^	31	1.97	0.75	32	2.34	0.59	Yes (lower), *p* < 0.05
Rodrigue et al. (2018)—weight subscale^d^	31	2.13	0.77	32	2.50	0.71	Yes (lower), *p* < 0.05
Rodrigue et al. (2018)—attribution subscale^d^	31	2.26	0.63	32	2.18	0.53	No, *p* > 0.05
Waldorf et al. (2019)—total score^d^	24	2.07	0.56	24	3.12	0.38	Yes (lower), *p* < 0.001
Rosenberg self‐esteem scale (RSES; Rosenberg, 1965)							
Rodrigue et al. (2018)—total score^a^	31	21.23	5.13	32	22.78	4.23	No, *p* > 0.05
Other instruments							
Difficulties in emotion regulation scale (DERS; Gratz & Roemer, 2004)							
Murray, Maguire, et al. (2012)—emotional awareness subscale^a^	1	26.00	n/a	n/a	n/a	n/a	n/a
Murray, Maguire, et al. (2012)—emotion regulation strategies subscale^a^	1	34.00	n/a	n/a	n/a	n/a	n/a
Murray, Maguire, et al. (2012)—emotional clarity subscale^a^	1	18.00	n/a	n/a	n/a	n/a	n/a
Murray, Maguire, et al. (2012)—goal‐directed behaviour subscale^a^	1	16.00	n/a	n/a	n/a	n/a	n/a
Murray, Maguire, et al. (2012)—acceptance of emotions subscale^a^	1	9.00	n/a	n/a	n/a	n/a	n/a
Murray, Maguire, et al. (2012)—impulse control subscale^a^	1	4.00	n/a	n/a	n/a	n/a	n/a
Multidimensional perfectionism scale (MPS; Frost et al., 1990)							
Rodrigue et al. (2018)—total score^a^	31	85.48	18.35	32	75.03	16.07	Yes (higher), *p* < 0.05
Obsessive‐compulsive personality scale (OCPS; Lazare et al., 1966)							
Rodrigue et al. (2018)—total score^a^	31	24.90	5.69	32	24.88	4.16	No, *p* > 0.05
Pathological narcissism inventory (PNI; Pincus et al., 2009)							
Rodrigue et al. (2018)—grandiose subscale^d^	31	3.97	0.63	32	3.40	0.76	Yes (higher), *p* < 0.05
Rodrigue et al. (2018)—vulnerable subscale^d^	31	3.36	0.62	32	2.92	0.73	Yes (higher), *p* < 0.05
Rodrigue et al. (2018)—total score^d^	31	3.62	0.51	32	2.13	0.61	Yes (higher), *p* < 0.05

^a^ score involves sum of all items in a subscale or across all subscales.

^b^ score at baseline assessment.

^c^ scores prior to a period of religious fasting.

^d^ score involves average of all items in a subscale or across all subscales.

^e^ control group includes people with diagnosed body dysmorphic disorder (BDD).

^f^ summed scores were used rather than an average score.

^g^ anxiety subscale omitted due to poor internal consistency (Cronbach's alpha = .52).

## 2 METHOD

The method for this study has been described elsewhere in more detail [Martenstyn, Maguire, et al., 2022a].

### 2.1 Participants

#### 2.1.1 Inclusion criteria

Eligible participants were required to: (a) be aged above 18 years, (b) report thinking about not being muscular enough at least once daily on a pre‐screening form, and (c) meet diagnostic criteria for MD based on a semi‐structured diagnostic interview developed by this research team in line with both the Pope et al. (1997) and DSM‐5 diagnostic criteria for MD (American Psychiatric Association, 2013). People of all genders and sexes were eligible for inclusion. The final sample of questionnaire responders comprised 26 adults with diagnosed MD (see Table 2).

**TABLE 2:** Sample characteristics.

Characteristic	n	%
Sex		
Male	25	96%
Female	1	4%
Location		
North America	14	54%
Europe	5	19%
United Kingdom	4	15%
East Asia	2	8%
Oceania	1	4%
Age		
18–19 years	2	8%
20–29 years	18	69%
30–39 years	6	23%
Competed or plans to compete in bodybuilding		
Yes	4	15%
No	22	85%
Recruitment source		
r/Moreplatesmoredates	9	35%
r/Swoleacceptance	8	31%
r/Naturalbodybuilding	7	27%
r/Steroids	1	4%
Referral from friend	1	4%

#### 2.1.2 Recruitment

Participants were recruited in two stages from October 2021 to February 2022. Stage one involved asking gyms located in Sydney and Melbourne to share our study advertisements via a combination of flyers, email, and social media posts. Stage two involved posting our study advertisements on 10 reddit forums related to body dysmorphia, bodybuilding, steroids, and men's health. Of the 169 people who signed up for a diagnostic assessment interview, 67 attended, 29 met formal criteria for MD, and 26 completed all questionnaires. Participants were compensated financially via e‐gift cards for their involvement in all aspects of the study.

### 2.2 Materials

Two semi‐structured interview guides were developed for this study: (a) a diagnostic assessment interview which addressed each individual criterion across both the Pope et al. (1997) and DSM‐5 diagnostic criteria for MD (American Psychiatric Association, 2013), and (b) a longer interview exploring the exercise routines and attitudes towards exercise of eligible participants. The complete list of questions included in both interview guides has been presented elsewhere [Martenstyn, Maguire, et al., 2022a].

### 2.3 Procedure

#### 2.3.1 Interviews

Prospective participants who responded to a study advertisement were taken to a pre‐screening webpage, and those who disclosed thinking about not being muscular enough at least once daily and were willing to provide informed consent were guided to book in a diagnostic interview using Calendly software. All diagnostic interviews were conducted by the first author via Zoom, lasted an average of 30 min, and were audio recorded to be reviewed by the senior author, who has extensive experience working with MD samples. Both the first and senior authors needed to agree that a participant met MD diagnostic criteria to be eligible for study inclusion. Diagnostic agreement at first assessment was high (>80%) between the two reviewers and there were no instances of disagreement at second assessment.

#### 2.3.2 Questionnaires

Along with attending the longer exercise interview, eligible participants were asked to complete a battery of questionnaires assessing MD symptomology, compulsive exercise, and disordered eating (see Table 3). We collected data on two MD questionnaires: (a) the MASS as it is one of the most commonly used questionnaires to assess the severity of MD symptoms (Badenes‐Ribera et al., 2019; Sandgren & Lavallee, 2018) and (b) the Muscle Dysmorphia Questionnaire (MDQ; Grieve & Shacklette, 2012), an unpublished instrument which we felt provided a comprehensive overview of the core MD behaviours identified in our recent qualitative study [Martenstyn, Maguire, et al., 2022a, 2022b]. We also administered the MDDI but excluded it from statistical analysis due to a clerical error in the formatting of response options. Rather than the original 5‐point Likert scale ranging from 1 (never) to 5 (always), a 5‐point Likert scale ranging from 1 (strongly disagree) to 5 (strongly agree) was mistakenly assigned, meaning the data structure for the MDDI was no longer ordinal in nature. Although compulsive exercise is a core feature of MD, there are no questionnaires specifically assessing the exercise behaviour of people with MD (Martenstyn, Aouad, et al., 2022). As such, we administered two questionnaires frequently used to assess compulsive exercise in eating disorder populations: (a) the Compulsive Exercise Test (CET; Taranis et al., 2011) and (b) the Exercise Dependence Scale (EDS; Hausenblas & Downs, 2002). Participants also completed two measures of disordered eating: (a) a widely used measure of thinness‐oriented disordered eating (EDE‐Q; Fairburn & Beglin, 1994) and (b) the Muscularity Oriented Eating Test (MOET; Murray et al., 2019), a recently developed questionnaire that captures disordered eating behaviours and cognitions specific to the pursuit of muscularity. Participants completed all questionnaires online via Qualtrics. Further details regarding the scoring and psychometric characteristics of each questionnaire are outlined in the Supplementary materials section.

**TABLE 3:** List of questionnaires administered in the current study.

Questionnaire	Subscales	Author	Items
Muscle dysmorphia			
Muscle appearance satisfaction scale (MASS)	1. Bodybuilding dependence 2. Muscle checking 3. Substance use 4. Injury 5. Muscle satisfaction	Mayville et al. (2002)	19
Muscle dysmorphia questionnaire (MDQ)	Unspecified factor structure	Grieve and Shacklette (2012)	34
Compulsive exercise			
Compulsive exercise test (CET)	1. Avoidance and rule‐driven behaviour 2. Weight control exercise 3. Mood improvement 4. Lack of exercise enjoyment 5. Exercise rigidity	Taranis et al. (2011)	24
Exercise dependence scale (EDS)	1. Tolerance 2. Withdrawal 3. Intention effect 4. Lack of control 5. Time 6. Reductions in other activities 7. Continuance	Hausenblas and Downs (2002)	21
Disordered eating			
Eating disorder examination questionnaire (EDE‐Q)	1. Restraint 2. Eating concern 3. Shape concern 4. Weight concern	Fairburn and Beglin (1994)	28
Muscularity‐oriented eating test (MOET)	Single factor structure	Murray et al. (2019)	15

### 2.4 Data analysis

**FIGURE 1:**
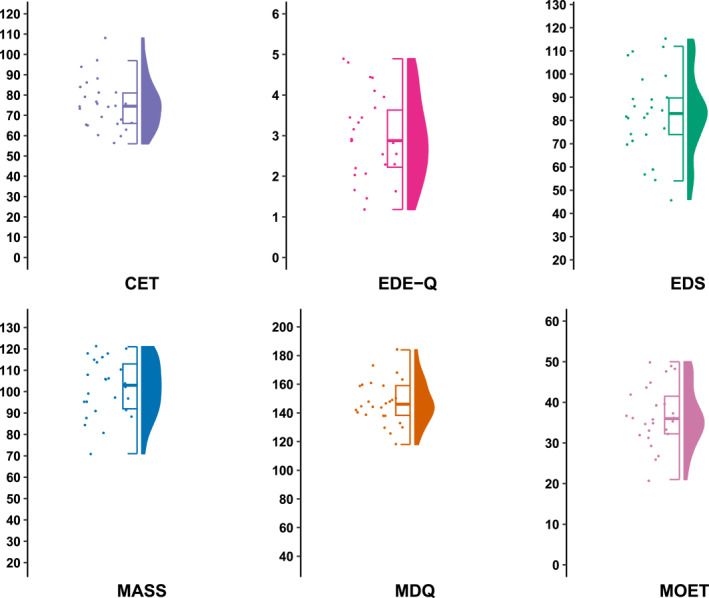
Raincloud plots visualising the distribution of total scores for study measures. The left‐hand side of each plot displays a half boxplot with individual datapoints overlayed. The top and bottom lines (i.e., hinges) of the plot denote the first and third quartile of scores, and the midline indicates the median. The whiskers of each plot extent to 1.5x the inter‐quartile range. The right‐hand side of each plot displays the frequency of total scores for each measure, with wider sections denoting more frequent responses. Large transparent circles represent outliers.

Descriptive statistics (mean, median, standard deviation, and range) were calculated for both total and subscale scores for each questionnaire. The distribution of scores for each questionnaire has been presented using Raincloud plots (Figure 1). Where clinical cut‐off scores have been proposed for a given questionnaire, we reported the percentage of participants whose total score exceeded that clinical cut‐off score. Internal consistency was assessed using Cronbach's alpha (*α*), with values ≥ 0.70 considered “acceptable” according to established criteria (Bland & Altman, 1997). Bivariate correlations between study measures were also calculated using Pearson's correlation coefficient. Based on existing guidelines for behavioural sciences research, we defined correlation coefficients (*r*) above 0.70 as “strong”, between 0.50 and 0.70 as “moderate”, and between 0.30 and 0.50 as “weak” (Hinkle et al., 2003). Statistical analyses were performed using the Statistical Package for the Social Sciences (SPSS) Version 28.

## 3 RESULTS

The final sample of questionnaire responders consisted of 26 adults with diagnosed MD (25 cisgender males, one cisgender female), most of whom (54%) were located in North America and aged between 20 and 29 years (69%). Participant characteristics are outlined in Table 2. Descriptive statistics for each questionnaire are presented in Table 4.

**TABLE 4:** Descriptive statistics for questionnaires administered in the current study.

	Mean	Median	SD	Possible range	Observed range	α
Muscle dysmorphia instruments						
Muscle appearance satisfaction scale (MASS; Mayville et al., 2002)						
Bodybuilding dependence subscale^a^	28.19	28.50	3.83	5–35	20–35	0.43
Muscle checking subscale^a^	20.00	19.00	4.90	4–28	10–28	0.65
Substance use subscale^a^	19.08	19.00	5.40	4–28	7–28	0.54
Injury risk subscale^a^	16.00	16.50	3.90	3–21	6–21	0.52
Muscle satisfaction subscale^a^	18.31	18.00	2.68	3–21	10–21	0.75
Total score^a^	101.58	103.00	13.22	19–133	71–121	0.74
Muscle dysmorphia questionnaire (MDQ; Grieve & Shacklette, 2012)						
Total score^a^	147.54	146.00	15.21	34–204	118–184	0.77
Compulsive exercise instruments						
Compulsive exercise test (CET; Taranis et al., 2011)						
Avoidance and rule‐driven behaviour subscale^a^	28.12	27.00	6.62	0–40	18–40	0.79
Weight control subscale^a^	13.62	14.50	5.19	0–25	4–21	0.69
Mood improvement subscale^a^	18.69	19.50	4.34	0–25	9–25	0.78
Lack of exercise enjoyment subscale^a^	3.42	3.00	3.10	0–15	0–10	0.69
Exercise rigidity subscale^a^	11.58	12.00	2.30	0–15	7–15	0.21
Total score^a^	75.42	74.50	12.33	0–120	56–108	0.75
Exercise dependence scale (EDS; Hausenblas & Downs, 2002)						
Tolerance subscale^a^	13.19	13.00	3.31	3–18	6–18	0.70
Withdrawal subscale^a^	12.15	13.00	3.50	3–18	6–18	0.83
Intention effects subscale^a^	11.92	12.00	4.10	3–18	4–18	0.85
Lack of control subscale^a^	12.35	13.00	3.73	3–18	5–18	0.85
Time subscale^a^	12.35	13.00	3.42	3–18	6–18	0.83
Reduction in other activities subscale^a^	10.12	10.50	2.86	3–18	4–16	0.47
Continuance subscale^a^	10.92	12.00	4.26	3–18	3–18	0.90
Total subscale^a^	83.00	83.00	17.69	21–126	46–115	0.91
Eating psychopathology instruments						
Eating disorder examination questionnaire (EDE‐Q 6.0; Fairburn & Beglin, 1994)						
Dietary restraint subscale^b^	3.22	3.60	1.66	0–6	0.20–5.80	0.75
Eating concern subscale^b^	1.92	1.90	1.09	0–6	0.20–4.20	0.52
Shape concern subscale^b^	3.68	3.69	1.18	0–6	1.75–5.38	0.76
Weight concern subscale^b^	3.04	3.00	1.11	0–6	0.60–5.40	0.44
Total score^b^	2.96	2.88	1.05	0–6	1.18–4.89	0.87
Muscularity oriented eating test (MOET; Murray et al., 2019)						
Total score^a^	36.77	36.00	7.43	0–60	21–50	0.65

^a^ score involves sum of all items in a subscale or across all subscales.

^b^ score involves average of all items in a subscale or across all subscales.

### 3.1 Muscle dysmorphia questionnaires

The mean total score on the MASS was 101.58 (SD = 13.22) with a maximum possible score of 133. The mean total score on the MDQ was 147.54 (SD = 15.21) out of a maximum possible score of 204. No clinical cut‐offs have been proposed for these two instruments.

**TABLE 5:** Bivariate correlation matrix for study measures.

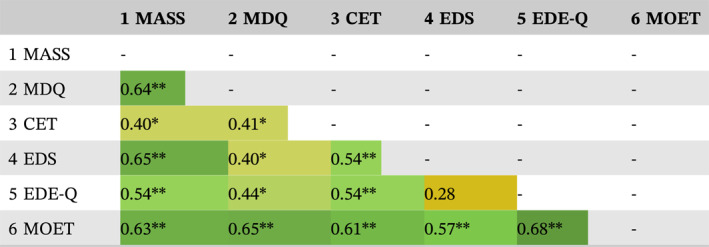

*Note*: *p < 0.05; **p < 0.01.

Abbreviations: CET, Compulsive Exercise Test; EDE‐Q, Eating Disorder Examination‐Questionnaire; EDS, Exercise Dependence Scale; MASS, Muscle Appearance Satisfaction Scale; MDQ, Muscle Dysmorphia Questionnaire; MOET, Muscularity Oriented Eating Test.

### 3.2 Compulsive exercise questionnaires

The mean total score on the CET was 75.42 (SD = 12.33) out of a maximum possible score of 120. All participants scored above the CET clinical cut‐off score of 15 proposed by Meyer et al. (2016) in women with eating disorders. On the EDS, the mean total score was 83.00 (SD = 17.69) with a maximum possible score of 126.

### 3.3 Disordered eating questionnaires

The mean total score on the EDE‐Q was 2.96 (SD = 1.05) with a maximum possible score of 6.00. Most participants (85%) scored above the clinical cut‐off score of 1.68 purported to delineate adult males with and without an eating disorder (Schaefer et al., 2018). Fifteen participants (58%) scored above the clinical cut‐off score of 2.80 proposed to denote an eating disorder in adult females (Mond et al., 2008). On the MOET, the mean total score was 36.77 (SD = 7.43) out of a maximum possible score of 60.

### 3.4 Bivariate correlations

Bivariate correlations revealed significant positive associations between almost all study measures (Table 5). Moderate positive correlations were identified between the following questionnaires: MASS and MDQ (*r* = 0.64), EDS (*r* = 0.65), EDE‐Q (*r* = 0.54), and MOET (*r* = 0.63); MDQ and MOET (*r* = 0.65); CET and EDS (*r* = 0.54), EDE‐Q (*r* = 0.54), and MOET (*r* = 0.61); EDS and MOET (*r* = 0.57); and EDE‐Q and MOET (*r* = 0.68).

## 4 DISCUSSION

To date, clinical norms have not been established for any questionnaires assessing the core cognitive‐behavioural features of MD. To add to the small base of literature that has published questionnaire data from a clinical MD sample, we presented descriptive statistics for six questionnaires that were administered in a sample of 26 adults who met formal diagnostic criteria for MD. We found that scores across a range of instruments assessing MD symptomology, compulsive exercise, and disordered eating were high in absolute terms and broadly consistent with those reported in past cross‐sectional descriptive studies and individual case reports. While we did not recruit a control group in this study, scores for our MD participants were substantially higher than those reported in regular exercisers without MD used as control groups in past studies (see Table 1). Scores across almost all questionnaires were significantly positively correlated with each other. Overall, these results suggest that adults with diagnosed MD score similarly on questionnaires appraising core features of the disorder.

As expected, participants in our sample scored high on both measures of general MD symptomology. On the MASS, individual subscale scores were very similar between our study and the sole study that administered the MASS in a clinical MD sample (Cafri et al., 2008). While the MDQ has not been psychometrically validated, the high mean score of 147.54 and high minimum score (118 out of a possible 204) suggests that the instrument is aptly capturing core cognitions and behaviours involved in MD. Although we were unable to report on the MDDI due to a clerical issue with the formatting of response options, our literature review indicates a narrow cluster of both total and subscale scores in past cross‐sectional descriptive and case studies (e.g., Blashill et al., 2020; Murray et al., 2012; Murray et al., 2011). More research is needed to develop clinical norms for MD‐specific questionnaires and determine whether diagnostic accuracy is enhanced by administering questionaries assessing MD symptomology in conjunction with a clinical interview.

Participants also scored high on instruments assessing compulsive exercise. On the CET, all participants scored substantially above the clinical cut‐off score of 15 used in past research to classify females with an eating disorder as “compulsive exercisers” (Meyer et al., 2016). Mean total and subscale scores were also in line with results from the only study that administered the CET in adults with diagnosed MD and were generally comparable with scores in this same study for adult males with anorexia nervosa (Murray et al., 2012). No prior studies have administered the EDS in people with diagnosed MD. However, the high subscale scores reported in this study are broadly aligned with findings from a recent qualitative study that people with MD frequently exercise to avoid withdrawal symptoms, experience notable distress if forced to miss or shorten a workout, and sacrifice social and/or occupational activities to maintain their workout schedule [Martenstyn, Maguire, et al., 2022b]. More research is needed to better understand the characteristics of compulsive exercise in MD and whether MD‐specific questionnaires for compulsive exercise—as opposed to those developed primarily for people with eating disorders—are warranted.

Our results indicate that people with clinical MD have high levels of both thinness‐oriented and muscularity‐specific disordered eating. On the EDE‐Q, most participants scored above clinical cut‐off scores proposed to indicate significant eating disorder psychopathology in both males (Schaefer et al., 2018) and females (Mond et al., 2008). Total and subscale EDE‐Q scores were also consistent with the only previous study that administered the EDE‐Q in a clinical MD sample, although we reported slightly higher scores on the eating concern subscale in our sample (Murray et al., 2012). Although no studies have administered the MOET in a clinical MD sample, our mean total score of 36.8 was substantially above the mean scores of 11.3 and 13.7 reported in non‐clinical male university student samples in the original MOET validation studies (Murray et al., 2019). While more questionnaire data is needed to generate clinical norms on disordered eating questionnaires, our results support recent qualitative findings that people with MD have highly restrictive eating routines and base their food intake primarily around shape and weight concerns [Martenstyn, Maguire, et al., 2022a].

Strengths of the current study include a geographically diverse sample, use of multiple raters to determine MD diagnostic status, and transparent publication of a broad range of descriptive statistics and Raincloud plots. Limitations of this study include limited female representation, the absence of questionnaires assessing other clinically relevant aspects of MD (e.g., body checking or self‐esteem), and that we did not administer the questionnaires in a healthy or subclinical comparison group (e.g., adult bodybuilders not meeting diagnostic criteria for MD).

Overall, this study provides useful questionnaire data for a sample of 26 adults with diagnosed MD, particularly for instruments that have not been previously administered in a clinical MD sample (e.g., MDQ, EDS, and MOET). Scores on the other three questionnaires (MASS, CET, and EDE‐Q) were broadly consistent with those reported in past studies. Scores across all questionnaires were broadly positively correlated. Although clinical cut‐off scores have not been validated specifically for adults with MD, it was notable that the extent to which participant scores exceeded proposed cut‐off scores (albeit for other illness groups like eating disorders) was much larger for the CET (compulsive exercise) than the EDE‐Q (eating disorder psychopathology). This finding arguably supports earlier conceptualisations of MD that placed compulsive exercise as the primary concern and disordered eating as a secondary concern (Pope et al., 1997, 2000). Overall, more research with clinical MD samples is needed to generate precise clinical norms. We stress that questionnaire scores should not be used in isolation to infer whether a person has MD, but may represent a useful adjunct to a comprehensive clinical interview.

## CONFLICT OF INTEREST STATEMENT

No conflicts of interest to disclose.

## Supporting information

Supporting Information S1

## Data Availability

Data files and source materials are available from the corresponding author upon request.
