# Supplementary material for: A descriptive analysis of scoring patterns on clinically relevant questionnaires in 26 adults with diagnosed muscle dysmorphia
Source: Eur Eat Disord Rev. 2023 Jun 19;31(6):737–51. doi: 10.1002/erv.3001 (PMC10947593; doi:10.1002/erv.3001)
Supplement: Supplementary file 1 — Supporting Information S1 [file ERV-31-737-s001.docx]

**Supplementary Materials – Questionnaire Information**

**Muscle Appearance Satisfaction Scale (MASS)**

The Muscle Appearance Satisfaction Scale (MASS) is a 19-item self-report measure assessing the severity of MD symptoms (Mayville et al., 2002). Items are scored on a 7-point Likert scale ranging from 1 (strongly disagree) to 7 (strongly agree) with higher scores indicative of more severe MD symptoms. The MASS can be separated into five subscales: (1) bodybuilding dependence (e.g., “I often feel like I am addicted to working out with weights”), (2) muscle checking (e.g., “I often spend a lot of time looking at my muscles in the mirror”), (3) substance use (e.g., “I often spend money on muscle-building supplements”), (4) injury risk (e.g., “I often ignore a lot of physical pain while I am lifting to get bigger”), and (5) muscle satisfaction (e.g., “I am satisfied with the size of my muscles”). Items on the MASS are summed to derive a total score ranging from 19-133. Three items on the muscle satisfaction scale (items 1, 4, and 14) are reversed scored. The scale has demonstrated adequate reliability and validity in adult American recreational weightlifters (Mayville et al., 2002) and Irish and British adult males (Ryan & Morrison, 2010). While several researchers have adapted the MASS to a 5-point Likert scale (e.g., Jin et al., 2015; Ryan & Morrison, 2010), we opted to retain the original 7-point structure for this study. Cronbach’s alpha for the MASS in this study was acceptable (α = .74).

**Muscle Dysmorphia Questionnaire (MDQ)**

The Muscle Dysmorphia Questionnaire (MDQ) is a 34-item self-report questionnaire that assesses symptoms of MD. Items are scored on a 6-point Likert scale ranging from 1 (strongly disagree) to 6 (strongly agree) with higher scores indicating greater MD symptomology. No factor structure has been proposed for the MDQ. Sample questions include: “I am inclined to continue to work out when I am sick” and “When I see other muscular men, it makes me feel badly about my body shape or size”. Items on the MDQ are summed to calculate a total score ranging from 34-204. Items 12, 16, and 21 are reverse scored. While the original validation study was not published, subsequent studies in non-clinical samples have reported high internal consistencies (α = .86-90) and strong correlations (*r* = .81) with the Muscle Dysmorphia Inventory (Grieve & Shacklette, 2012; Lowe, 2014). Internal consistency for the MDQ in the current study was acceptable (α = .77).

**Compulsive Exercise Test (CET)**

The Compulsive Exercise Test (CET) is a 24-item self-report measure designed to assess the core cognitive, behavioural, and emotional features of compulsive exercise (Taranis et al., 2011). Items are scored on a 6-point Likert scale ranging from 0 (never true) to 5 (always true). Higher scores indicate greater levels of compulsive exercise. The CET comprises five subscales, including: (1) avoidance and rule-driven behaviour (e.g., “If I miss an exercise session, I will try and make up for it when I next exercise”), (2) weight control exercise (e.g., “if I feel I have eaten too much, I will do more exercise”), (3) mood improvement (e.g., “I feel happier and/or more positive after I exercise”), (4) lack of exercise enjoyment (e.g., “I find exercise a chore”), and (5) exercise rigidity (e.g., “my weekly pattern of exercise is repetitive”). Items on the CET are summed to derive a total score ranging from 0-120. Items 8 and 12 are reverse scored. A clinical cut-off score of 15 has been purported to indicate significant levels of compulsive exercise in people with an eating disorder (Meyer et al., 2016). The CET has demonstrated good psychometric properties across studies sampling individuals both with and without an eating disorder (Harris et al., 2020; Taranis et al., 2011). Internal consistency for the CET in the current study was acceptable (α = .75).

**Exercise Dependence Scale (EDS)**

The Exercise Dependence Scale (EDS) is a 21-item self-report questionnaire assessing exercise dependence as operationalised based on the DSM-IV criteria for substance dependence (Hausenblas & Downs, 2002). Items are scored on a 6-point Likert scale ranging from 1 (never) to 6 (always) with higher scores indicating greater levels of exercise dependence. The EDS has seven subscales: (1) withdrawal effects (e.g., “I exercise to avoid feeling anxious”), (2) continuance (e.g., “I exercise when injured”), (3) tolerance (e.g., “I continually increase my exercise duration to achieve the desired effects/benefits), (4) lack of control (e.g., “I am unable to reduce how often I exercise”), (5) reduction in other activities (e.g., “I think about exercise when I should be concentrating on school/work”), (6) time (e.g., “I spend a lot of time exercising”), and (7) intention effects (e.g., “I exercise longer than I plan”). Items on the EDS are summed to calculate a total score ranging from 21-126. The EDS has demonstrated good validity and reliability across both clinical and non-clinical samples (Hausenblas & Downs, 2002; Mónok et al., 2012). Internal consistency for the EDS in the current study was excellent (α = .91).

**Eating Disorder Examination-Questionnaire (Sixth Edition) (EDE-Q)**

The sixth and latest edition of the Eating Disorder Examination-Questionnaire (EDE-Q) is a 28-item self-report questionnaire that measures the primary cognitive and behavioural symptoms of an eating disorder (Fairburn & Beglin, 1994). Items are scored on a 7-point Likert scale ranging from 0 (no days) to 6 (every day). Higher scores are indicative of more severe eating disorder psychopathology. The EDE-Q has four subscales: (1) dietary restraint (e.g., “have you had a definite desire to have an empty stomach with the aim of influencing your shape or weight?”), (2) eating concern (e.g., “have you had a definite fear of losing control over eating?”), (3) shape concern (e.g., “have you felt fat?”), and (4) weight concern (e.g., “have you had a strong desire to lose weight?”). As the number of items is not consistent between subscales, subscale scores are derived from the average score per item. The total EDE-Q score is calculated as the average of the four subscale scores. Several clinical cut-off scores have been purported to delineate clinical eating disorder symptoms, including 2.80 in adult females (Mond et al., 2008) and 1.68 in adult males (Schaefer et al., 2018). The EDE-Q has demonstrated excellent psychometric properties across both clinical and non-clinical eating disorder samples (Berg et al., 2012). Cronbach’s alpha for the EDE-Q in this study was good (α = .87).

**Muscularity-Oriented Eating Test (MOET)**

The Muscularity-Oriented Eating Test (MOET) is a 15-item self-report questionnaire that assesses disordered eating cognitions and behaviours associated with the pursuit of muscularity (Murray et al., 2019). Items are scored on a 5-point Likert scale ranging from 0 (never true) to 4 (always true). Higher scores indicate greater levels of muscularity-oriented disordered eating. The MOET has a single factor structure and items can be summed to derive a total score ranging from 0-60 (Murray et al., 2019). The MOET has demonstrated good psychometric properties in undergraduate college males (Murray et al., 2019). Internal consistency for the MOET in the present study was moderate (α = .65).

**References**

Berg, K. C., Peterson, C. B., Frazier, P., & Crow, S. J. (2012). Psychometric evaluation of the eating disorder examination and eating disorder examination-questionnaire: a systematic review of the literature. *Int J Eat Disord*, *45*(3), 428-438. <https://doi.org/10.1002/eat.20931>

Fairburn, C. G., & Beglin, S. J. (1994). Assessment of eating disorders: interview or self-report questionnaire? *Int J Eat Disord*, *16*(4), 363-370.

Grieve, F. G., & Shacklette, M. D. (2012). Brief report on men's bodies & mood: Correlates between depressive symptoms & muscle dysmorphia symptoms. *North American Journal of Psychology*, *14*, 563-568.

Harris, A., Hay, P., & Touyz, S. (2020). Psychometric properties of instruments assessing exercise in patients with eating disorders: a systematic review. *Journal of Eating Disorders*, *8*(1), 45. <https://doi.org/10.1186/s40337-020-00315-2>

Hausenblas, H. A., & Downs, D. S. (2002). How much is too much? The development and validation of the exercise dependence scale. *Psychology & Health*, *17*(4), 387-404. <https://doi.org/10.1080/0887044022000004894>

Jin, X., Jin, Y., Zhou, S., Li, X., Yang, S. N., Yang, D., Nieuwoudt, J. E., & Yao, J. (2015). The Muscle Appearance Satisfaction Scale: A factorial analysis of validity and reliability for its use on adult Chinese male weightlifters. *Body Image*, *14*, 94-101. <https://doi.org/10.1016/j.bodyim.2015.04.004>

Lowe, A. B. (2014). *Objective Measures and Insight Assessments of Muscle Dysmorphia* (Publication Number Paper 1397) [Masters Theses & Specialist Projects, Western Kentucky University]. <https://digitalcommons.wku.edu/cgi/viewcontent.cgi?article=2401&context=theses>

Mayville, S. B., Williamson, D. A., White, M. A., Netemeyer, R. G., & Drab, D. L. (2002). Development of the Muscle Appearance Satisfaction Scale: a self-report measure for the assessment of muscle dysmorphia symptoms. *Assessment*, *9*(4), 351-360. <https://doi.org/10.1177/1073191102238156>

Meyer, C., Plateau, C. R., Taranis, L., Brewin, N., Wales, J., & Arcelus, J. (2016). The Compulsive Exercise Test: confirmatory factor analysis and links with eating psychopathology among women with clinical eating disorders. *J Eat Disord*, *4*, 22. <https://doi.org/10.1186/s40337-016-0113-3>

Mond, J. M., Myers, T. C., Crosby, R. D., Hay, P. J., Rodgers, B., Morgan, J. F., Lacey, J. H., & Mitchell, J. E. (2008). Screening for eating disorders in primary care: EDE-Q versus SCOFF. *Behav Res Ther*, *46*(5), 612-622. <https://doi.org/10.1016/j.brat.2008.02.003>

Mónok, K., Berczik, K., Urbán, R., Szabo, A., Griffiths, M. D., Farkas, J., Magi, A., Eisinger, A., Kurimay, T., Kökönyei, G., Kun, B., Paksi, B., & Demetrovics, Z. (2012). Psychometric properties and concurrent validity of two exercise addiction measures: A population wide study. *Psychology of Sport and Exercise*, *13*, 739-746. <https://doi.org/10.1016/j.psychsport.2012.06.003>

Murray, S. B., Brown, T. A., Blashill, A. J., Compte, E. J., Lavender, J. M., Mitchison, D., Mond, J. M., Keel, P. K., & Nagata, J. M. (2019). The development and validation of the muscularity-oriented eating test: A novel measure of muscularity-oriented disordered eating. *Int J Eat Disord*, *52*(12), 1389-1398. <https://doi.org/10.1002/eat.23144>

Ryan, T. A., & Morrison, T. G. (2010). Psychometric properties of the Muscle Appearance Satisfaction Scale among Irish and British men. *Body Image*, *7*(3), 246-250. <https://doi.org/https://doi.org/10.1016/j.bodyim.2010.02.008>

Schaefer, L. M., Smith, K. E., Leonard, R., Wetterneck, C., Smith, B., Farrell, N., Riemann, B. C., Frederick, D. A., Schaumberg, K., Klump, K. L., Anderson, D. A., & Thompson, J. K. (2018). Identifying a male clinical cutoff on the Eating Disorder Examination-Questionnaire (EDE-Q). *Int J Eat Disord*, *51*(12), 1357-1360. <https://doi.org/10.1002/eat.22972>

Taranis, L., Touyz, S., & Meyer, C. (2011). Disordered eating and exercise: Development and preliminary validation of the compulsive exercise test (CET). *European Eating Disorders Review*, *19*(3), 256-268. <https://doi.org/10.1002/erv.1108>
